# Acute kidney injury in patients with paraquat intoxication; a case report and review of the literature

**DOI:** 10.15171/jrip.2016.43

**Published:** 2016-08-03

**Authors:** Afshin Safaei Asl, Peyman Dadashzadeh

**Affiliations:** ^1^Division of Nephrology, Department of Pediatrics, Guilan University of Medical Sciences, Rasht, Iran; ^2^Resident of Pediatrics , Guilan University of Medical Sciences, Rasht, Iran

**Keywords:** Paraquat, Poisoning, Suicide, Acute kidney injury

## Abstract

Paraquat and diquat are classified as bipyridyl compounds not only leads to acute organ damage, but also to a variety of complications. Patients with severe paraquat-induced poisoning may succumb to multiple organ failure involving the circulatory and respiratory systems. Deliberate self-poisoning with paraquat continues to be a major public health concern in many developing countries. At present there is no specific antidote to paraquat poisoning, hence the need to more focus on prevention and in cases of poisoning aggressive decontamination to prevent further absorption. This article presented a 12-year-old male with acute kidney injury following the ingestion of paraquat in suicidal attempt and serves to explore the complications associated with paraquat poisoning and current recommended treatment

**Implication for health policy/practice/research/medical education:**

Paraquat is a broad spectrum liquid herbicide associated with both accidental and intentional ingestion. Toxicity is usually seen following ingestion, and may range from mild to fulminant with the latter commonly proving fatal. In addition to intense local irritation of the mouth, oropharynx and esophagus, multiple organ (cardiac, respiratory, hepatic and renal) failure may occur, although pulmonary features predominate and are the usual cause of death. This article presented a 12-year-old male with acute kidney injury following the ingestion of paraquat in suicidal attempt that admitted and discussed the probable ways for effective management of the patient.

## Introduction

Paraquat (1, r-dimethyl-4, 4’-bipyridium dichloride), is classified as bipyridyl compounds. Paraquat poisoning is usually seen following ingestion of the poison. A high dose of paraquat or severe poisoning has a poor prognosis (1,2). Paraquat as an applicable herbicide and relatively inexpensive has favorable environmental characteristics that was made for the first time in 1882 and has been used as an herbicide since 1955 (1). Paraquat has been widely used in much of the developing world, however thousands of individuals succumb due to paraquat intoxication every year in the developing world. Paraquat is a highly toxic compound and the fatality rate of paraquat is between 60% and 80% (2) because of lack of a specific antidote. A paraquat dose of 30 mg/kg may be fatal, which is equivalent to 8–10 mL of the 20% solution sold commercially (3). Paraquat has been shown to cause significant damage to organs, including the lung, liver, myocardium and kidneys, with the highest concentration of paraquat found in the lungs (4). The prognosis of patients with multiple organ failure caused by fulminant poisoning (>40 mg/paraquat ion per kg of body weight) is extremely dangerous and affected individuals may succumb within hours to a few days following ingestion (5,6). Since the first reported case of paraquat poisoning, studies have focused on the mechanisms and effects of combination therapies with various agents (7,8). Furthermore, strategies for the management of paraquat poisoning have focused on the modification of the toxic kinetics of the poison by either decreasing its absorption or improving its elimination (9). However, investigations on comprehensive strategies are rare. We previously identified a comprehensive treatment strategy against paraquat poisoning, termed the Qilu scheme (10). The present study indicates the potential and feasibility of the therapy for the treatment of paraquat poisoning. Informed consent was obtained from the patient and his parents. The study was approved by the ethics committee of the 17-Shahrivar hospital of Guilan University of Medical Sciences (Rasht, Iran).

## Case Presentation

**Figure 1:**
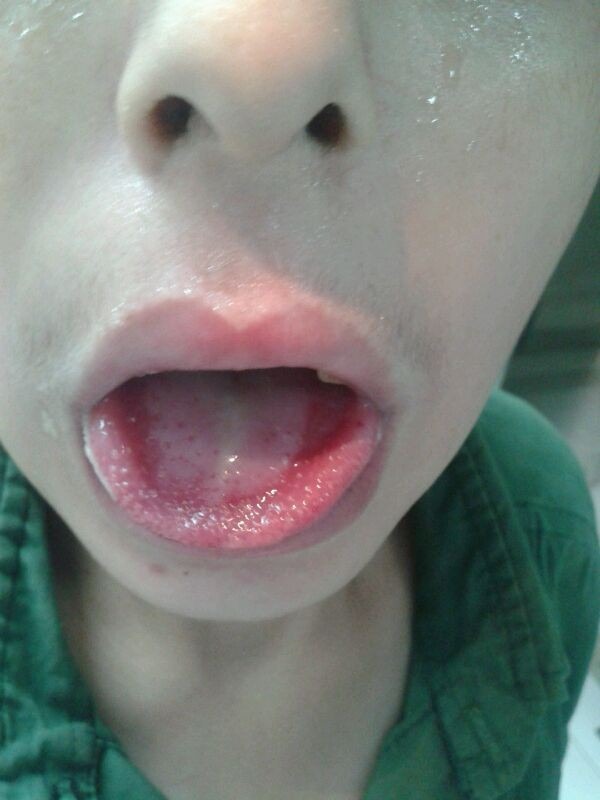
Oral ulcerations and edematous lip.

**Figure 2:**
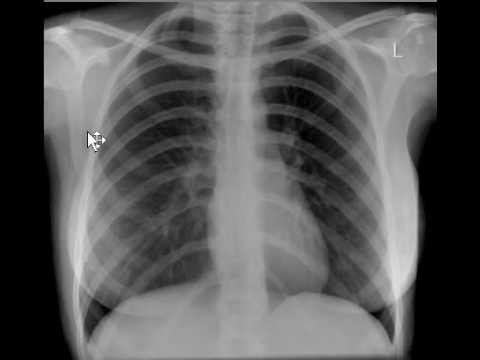
X-ray chest posteroanterior (PA) view showing mild bilateral patchy consolidation especially in hilum.

**Figure 3:**
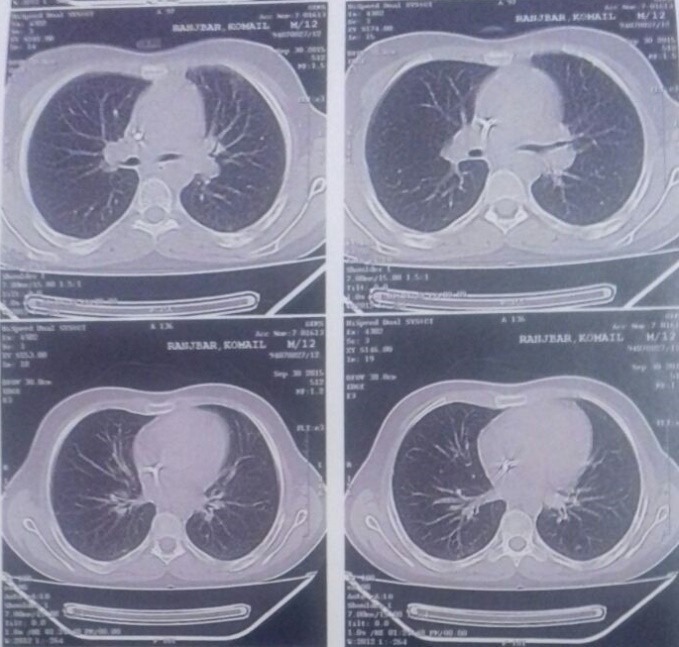
CT-scan of the thorax without contrast day 2 following paraquat poisoning showed sub- segmental collapses in the right costophrenic area.

A previously healthy 12-year-old male was referred to the emergency department 17-Shahrivar hospital 7 days after ingestion of an unknown quantity of a liquid. Following the ingestion, he had several episodes of vomiting that sometimes associated with bloody secretion. His appearance was pale. Relatives denied any episode of seizure. He experienced anorexia, lethargy and discomfort during last week. He was ill in appearance but arousal and able to speak, after following the parents to visit their public health city center and then the patient was referred to this center. On examination, he was drowsy but arousal, afebrile with heart rate 71/min, regular, BP 100/75 mm Hg, respiratory rate 18/min, and oxygen saturation (while breathing room air) 95%. His oral mucosa was congested and edematous. The following lesions consist exudative ulcerated tongue (Figure 1) and edematous lip were observed in the physical examination. Pupils were bilateral 2 mm and reacting to light. Both lung fields were clear on auscultationl.The pulse rate was 71 beats/min at a regular rhythm with no extra heart sounds or murmurs. On arrival the blood pressure of patient was 90/60 mm Hg. Overall the patient’s vital signs were normal, with a body temperature 37.7°C, pulse 71 beats/min, respiratory rate 18 breaths/min, blood pressure 90/60 mm Hg. There were no neurological symptoms, organomegaly, tachycardia or tachypnea. He received intravenous therapy (IV therapy). In the emergency, he received IV fluid and antiemetic (ondansetron) and ranitidine as supportive measure. Initial complete blood count, electrolytes, liver function tests, arterial blood gas, and serum amylase were within normal limit. His initial chest x-ray was normal (Figure 2). The patient had high serum urea (212 mg/dL) and creatinine (Cr) (8.6 mg/dL) on the day of admission that gradually reduced to normal after three cessation of dialysis. Blood and urine cultures were sterile. Other blood investigations including thyroid and liver functions were normal. Urine examination was normal and other finding was normal. The electrocardiogram (ECG) was normal. 2D echocardiography showed normal cardiac chambers with LVEF of 70%. Based on the typical poison-associated symptoms in the mouth and the gastrointestinal tract, computed tomography (CT) manifestations, significantly abnormal results in the main laboratory test results and the information provided by the patient and his relatives on admission, the patient was diagnosed with acute paraquat poisoning. Following admission to the hospital, other treatments such as an Ventolin nebulizer and also becotide spray, the patient was received antibiotic during admission including clindamycin 500 mg/TDS, cloxacillin 1 gm/QID, ceftriaxone 1.5 gm/BD and also pantoprazole 40 mg/TDS. The physician prescribed also drop nystatin for the oral lesions. Following therapy, the dynamic changes in the lung CT-scans are shown in Figure 3. The main laboratory test results are shown in Table 1. The kidney functions were seriously damaged 7 days following ingestion. Because of clinical signs and the continued increase in blood urea and creatinine hemodialysis was done for him. However, the kidney functions gradually recovered 9 days later completely (Table 1). However, the dialysis was started day 3 and after 3 cessation dialysis, kidney functions recovered. The patient discharged after 9 days with blood urea nitrogen (BUN) =19 mg/dL and Cr =1.3 mg/dL. He was followed a few weeks after discharge.

**Table 1:** Changes in the main parameters of blood, liver and kidney function test results for the patient 1, 2, 3, 4, 5, 6,7,8 and 9 days following paraquat ingestion

**Date**	**WBC**	**HGB**	**PLT**	**ESR**	**Cr**	**BUN**	**ALT**	**AST**	**NA**	**K**
Day 1	9200	12	245	20	‏10.5	212	11	16	139	3,4
Day 2	8600	11,8	249	19	‏11	223	10	17	151	3,4
Day 3	8400	11,1	240	16	4,5	114	11	16	145	3,8
Day 4	9000	11,3	255	14	3,2	63	10	13	140	3,8
Day 5	8800	11,2	266	14	2,3	3,4	9	14	145	3,7
Day 6	8900	11,1	277	11	2,3	34	10	13	142	3,8
Day 7	7900	11	275	7	2	30	11	14	141	3,7
Day 8	8000	10,8	280	5	1,8	28	10	14	140	3,6
Day 9	8900	10,4	249	3	1,3	19	10	15	138	3

Abbreviations: ALT, alanine aminotransferase; AST, aspartate aminotransferase; CR, creatinine; BUN, blood urea nitrogen; WBC, white blood cell; HGB, hemoglobin; PLT, platelet; ESR, erythrocyte sedimentation rate.

## Discussion

Paraquat is a non-selective herbicide that has been widely used in agriculture since the 1960s (11). Although it has been found to be safe for occupational use, paraquat poisoning usually occurring through ingestion of the poison either accidentally or intentionally in an attempt to suicide. Paraquat is banned or rarely used in the developed world; however, in developing countries paraquat continues to be used and paraquat poisoning remains a major cause of mortality among patients with acute poisoning (12). During acute paraquat poisoning, the level of drug concentration in lung is higher than plasma (13). Therefore, the primary cause of mortality in paraquat poisoning is often due to the lung involving mainly the alveolar epithelium, where it may initially lead to an acute alveolitis and later, pulmonary fibrosis (14). In addition to lung damage, paraquat ingestion has been shown to injure other organs, but to a lesser extent. Acute fulminant poisonings are characterized by with multiple organ failure (cardiac, respiratory, renal, hepatic, adrenal, pancreatic, and neurological) (15). Therefore, gastric lavage is mandatory to reduce the absorption of paraquat. Extracorporeal elimination, for example hemoperfusion (HP), is an effective modality that has been used clinically (11). Most of studies have showed the correlation between hemoperfusion and the final prognosis of paraquat poisoning. However, the results of these studies are controversial opinions have arisen on the therapeutic effects of HP in paraquat intoxication treatment (11,13). The prognosis for patients with severe paraquat poisoning due to multiple organ damage is critical. Despite various investigations into the mechanism of toxicity and the potential therapeutic treatments for paraquat poisoning, at present no specific therapy has been shown to affect the outcome in controlled clinical studies (12). Rapid and large increases in Cr are a common clinical presentation of severe paraquat poisoning and greatly exceed the value that predicted by a large decreases in glomerular filtration rate. In such cases the use of cystatin C is helpful. Early increase in cystatin C level in severe paraquat poisoning may be solution this problem. Loss of renal function contributes modestly to the large increases in creatinine following paraquat poisoning. The rapid rise in serum creatinine most probably represents increased production of creatine and creatinine to meet the energy demand following severe oxidative stress. Paraquat induced AKI needs to be evaluated using alternative kidney biomarkers of function and damage, which are more specific to kidney damage under these conditions (16).

In the present case study, a patient with suicidal paraquat poisoning was successfully treated using multi-target comprehensive therapy. The patient ingested a low dose of paraquat, resulting in severe kidney damage. The mechanism of paraquat toxicity has been previously investigated by Dinis-Oliveira et al (14). The patient was cured and discharged from hospital. However, further studies are required to determine an effective treatment against paraquat poisoning. In this case, vomiting the content of stomach readily after the ingestion of the poison, starting hemodialysis early after the admission, and use of other interventions help in the patient’s survival. The diagnosis of paraquat poisoning in the present study was mostly based on patient’s history and clinical examination. Serum levels of paraquat were not measured.

## Conclusion

Paraquat consumption is a rare agent of suicidal poisoning, resulting in very high morbidity and mortality. There is no specific antidote for paraquat poisoning and the mainstay of treatment is supportive. Acute kidney injury is the common complication of paraquat poisoning and needs to be well-known and treated promptly. Immediate and adequate interventions and hemodialysis have an undeniable and important role in survival of individuals after ingestion of paraquat.

## Authors’ contribution

All authors contributed equally to the study.

## Conflicts of interest

The author declared no competing interests.

## Ethical considerations

Ethical issues (including plagiarism, data fabrication, double publication) have been completely observed by authors.

## Funding/Support

None.

## References

[1] Wilks MF, Fernando R, Ariyananda PL, Eddleston M, Berry DJ, Tomenson JA (2008). Improvement in survival after paraquat ingestion following introduction of a new formulation in Sri Lanka. PLoS Med.

[2] Rose MS, Smith LL, Wyatt I (1974). Evidence for energy-dependent accumulation of paraquat into rat lung. Nature.

[3] Yang CJ, Lin JL, Lin-Tan DT, Weng CH, Hsu CW, Lee SY (2012). Spectrum of toxic hepatitis following intentional paraquat ingestion: analysis of 187 cases. Liver Int.

[4] Weng CH, Hu CC, Lin JL, Lin-Tan DZ, Huang WH, Hsu CW (2012). Sequential organ failure assessment score can predict mortality in patients with paraquat intoxication. PLoS One.

[5] Yoon SC (2009). Clinical outcome of paraquat poisoning. Korean J Intern Med.

[6] Bertram A, Haenel SS, Hadem J, Hoeper MM, Gottlieb J, Warnecke G (2013). Tissue concentration of PQ on day 32 after intoxication and failed bridge to transplantation by extracorporeal membrane oxygenation therapy. BMC Pharmacol Toxicol.

[7] Agarwal R, Srinivas R, Aggarwal AN, Gupta D (2007). Immunosuppressive therapy in lung injury due to paraquat poisoning: a meta-analysis. Singapore Med J.

[8] Suntres ZE (2002). Role of antioxidants in paraquat toxicity. Toxicology.

[9] Yeo CD, Kim JW, Kim YO, Yoon SA, Kim KH, Kim YS (2012). The role of pentraxin-3 as a prognostic biomarker in paraquat poisoning. Toxicol Lett.

[10] Castro R, Prata C, Oliveira L, Carvalho MJ, Santos J, Carvalho F (2005). Paraquat intoxication and hemocarboperfusion. Acta Med Port.

[11] Hampson EC, Pond SM (1988). Failure of haemoperfusion and haemodialysis to prevent death in paraquat poisoning. Med Toxicol Adverse Drug Exp.

[12] Lin JL, Lin-Tan DT, Chen KH, Huang WH, Hsu CW, Hsu HH (2011). Improved survival in severe paraquat poisoning with repeated pulse therapy of cyclophosphamide and steroids. Intensive Care Med.

[13] Sun ML, Ma DH, Liu M, Yu YX (2009). Successful treatment of paraquat poisoning by Xuebijing, an injection concocted from multiple Chinese medicinal herbs: a case report. J Altern Complement Med.

[14] Dinis-Oliveira RJ, Duarte JA, Sánchez-Navarro A, Remião F, Bastos ML, Carvalho F (2008). Paraquat poisonings: mechanisms of lung toxicity, clinical features, and treatment. Crit Rev Toxicol.

[15] Yu G, Kan B, Jian X, Wang J, Sun J, Song C (2014). A case report of acute severe paraquat poisoning and long-term follow-up. Exp Ther Med.

[16] Mohamed F, Endre Z, Jayamanne S, Pianta T, Peake P, Palangasinghe C (2015). Mechanisms underlying early rapid increases in creatinine in paraquat poisoning. PLoS One.

